# Podiatric surgery: friend or foe?

**DOI:** 10.1186/1757-1146-4-S1-I7

**Published:** 2011-05-20

**Authors:** Trevor D Prior

**Affiliations:** 1 Homerton University Hospital, London, E9 6SR, UK

## 

Podiatric surgery within the UK remains controversial in some quarters and, despite being well established within many National Health Service (NHS) centres, myth and rumour remain. All too often individual concerns and misreporting can foster negative publicity, yet the quality and standard of practice have facilitated continued development and establishment of services. This presentation will review the common areas of confusion and overview training, regulation, interaction with government and other medical bodies. The available evidence base will be reviewed in relation to patient satisfaction and outcomes / complications. If the profession as a whole is to continue to develop, integrated care pathways will be an essential component. The relevance of podiatric surgery to the profession as a whole and methods for integrating practice will be discussed.

